# Supplementary material for: Bioelectrical impedance analysis for early screening of upper limb subclinical lymphedema: A case–control study
Source: PLoS One. 2022 Sep 19;17(9):e0274570. doi: 10.1371/journal.pone.0274570 (PMC9484636; doi:10.1371/journal.pone.0274570)
Supplement: Attachment — Submitted filename: Response to Reviewers.docx [file pone.0274570.s001.docx]

Dear Dr. Lafrenie

Subject: Submission of revised paper PONE-D-22-02659

Thank you for your email dated 9 June 2022 enclosing the reviewers’ comments. We have carefully reviewed the comments and have revised the manuscript accordingly. Our responses are given in a point-by-point manner below. Changes to the manuscript are shown in a separate file labeled “Revised manuscript with Track Changes”.

We hope the revised version is now suitable for publication and look forward to hearing from you in due course.

Sincerely,

Shaoyong Wu

International lymphedema therapist

West China Hospital

**Response to Reviewer 1: Thank you for your review of our paper. We have answered each of your points below.**

1. **the authors should consider English language editing**

**Response:** Thanks very much for the suggestions from reviewer. Before resubmission, this manuscript edited and checked by a professional edition company (www.editage.com), which deals with English editing for non-native English researchers. We hope that our manuscript meets your requirements for publication now. We have also uploaded the proof of polishing the manuscript.

2. **it is not clear why menstruating is an exclusion criterion, could a BI assessment not be postponed for several days.**

**Response:** Lymphadenectomy is the primary cause of breast and upper extremity lymphedema in patients with breast cancer. Our study is an exploratory study. Exploratory objective are to evaluate potential influence of lymphadenectomy for breast cancer survivors. For females, testing during menstruation influence BI outcome. Due to this constraint, we choose to menstruating is an exclusion criterion.

In China, within 3 days post-consultation about treatment options, patients will receive first chemotherapy treatment. Patients spent these days in hospital. In fact, we chose the day before the data of first chemotherapy treatment to overcome the influence of chemotherapy. If BI assessment is postponed for several days, it's very likely that patients have received their first circle of chemotherapy. Chemotherapy is a major confounding factors in our study. Therefore, BI assessment could not be postponed for several days. In the end of the manuscript, we revised this paragraph**(Page 5, Paragraph 2)**.

3. **In comparison with BI a reference with a 2cm- difference based on circumefereces was used to define lymphedema. As demonstrated by Armer, in 2005 already, the 2 cm diff definition is a wrong definition to be used in the assessment of lymphedema.**

**Response:** Thank you for your suggestion. In the article, our essay mentions the standard of 2cm- difference based on circumference. This standard is outdated. Your suggestion pointed out exactly where we had gone wrong. I have revised the contents of this part.**(Page 7, Paragraph 3)** Thank you very much.

4. **For BI an approach of fasting and empty bladder is used => therefore this becomes a protocol that can only be used in the morning, limiting clinical application when patients are routinely seen on an ambulatory appointment.**

**Response:** Thank you, good question. Our study is an exploratory study. Hospitalized patients were recruited in order to facilitate longitudinal follow-up. It is also easy to implement and measure in practice. In fact, no one received initial chemotherapy as an outpatient in China. Moreover, The result of BI may be influenced by a multiplicity of different factors. To avoid possible influences of eating and drinking, an approach of fasting and empty bladder is used in our study. Further research regarding underlying influence of times(morning or afternoon) and meals is warranted. It would be a good idea to assess the feasibility of clinical application of outpatient. Thank you very much.

5. **the author state that a skilled therapist examined body composition, what was assessed and how?**

**Response:** Thank you for your question. I am very sorry that this part was not clear in the original manuscripts, I should have explain that our assessment will cost 5-10 minutes before measurement. A skilled therapist in our study assessed several aspects before measurement, such as whether the inclusion and exclusion criteria were met, whether skin changes occurred, whether limb pain or discomfort existed, whether the patient experienced upper extremity swelling or nonpitting edema, and whether the range of motion was restricted. BIA data were collected by a trained therapist. Implementation of guideline-recommended assessment in our study is appropriate for breast cancer patients. I have revised the content of this part. **(Page 6, Paragraph 1)**

6. **A major concern is that as a reference a healthy group was used. This healthy sample is significantly different from the patient sample. At least I expected a matched sample to draw any conclusions.**

**Response:** Thank you for your question. Our group member give serious consideration to this matter. We'd like to know the differences between groups in one of primary outcome measure（the ratio differences of impedance in 5KHz). However, there were some between-group demographic(age and BMI) differences. we establish the linear regression model by correcting demographic(age and BMI) differences. In fact, age and BMI were excluded variables. Only groups is stepwised entrying into the linear regression equations. These statistics show that there's no need for correction between groups using age and BMI. Therefore, we do not use the method of propensity score matching. For details, see below:

7. **It is unclear how an inter-limb difference can be calculated from a whole body BI. Additionally, a difference between dominant and non-dominant arms exist, this was found by the authors as well. How to deal with this in BI. The Methodology should elaborate on this, making section 3.5 better readable.**

**Response:** Thank you for your question. I am very sorry that this part was not clear in the original manuscripts, I should have explain that data collected form bioimpedance device includes five parts: left arm part, the right arm part, trunk part, left leg part and right leg part. All parts of data are numerical-type variables. Therefore, an inter-limbs difference can be calculated between the values of left and right arm in the same index.

In fact, the difference between both arms exists not only in the dominant and nondominant arms but also in the proximal and distal arms (according to the distance between the breast cancer location and arm). Our study showed that differences between arms exist in breast cancer survivors, which has not been suggested by previous research. As an exploratory study, the difference between both arms in the healthy group will be used to provide a benchmark that uses two standard deviation cut-offs for screening breast cancer survivors. Briefly, dominant arm is indistinguishable from the non-dominant arms. The concept(the difference between both arm) is better for us to assess the change of arm. Our study contrasted the difference between both arm in healthy group and in breast cancer participants.

Moreover, I have revised the content of Methodology, especially the content of section 3.5. **(Page 11, Paragraph 2)**

8. **Only 1 assessment was made to define subclinical lymphedema, this should be multiple times in follow up, as mentioned by the authors. Why not add prospective follow-up data to confirm the hypothesis that BI can be a diagnostic tool.**

**Response:** On the one hand, follow-up assessment may be affected because of follow-up treatment(chemotherapy and radiotherapy). The results of follow-up assessment does not represent this assessment. It is not necessary connection between follow-up and this measurement.On the other hand, this study formed part of a larger programme of research. Our research is a prospective cohort study. We will implement multiple follow-ups. It'll take 2 year, at the very least. That is the reason why we not add prospective follow-up data to confirm the hypothesis that BI can be a diagnostic tool.Therefore, your suggestion is the question which we will solve in the future.

However, BI is the diagnostic aids recommended by Clinical Practice Guideline From the Academy of Oncologic Physical Therapy of APTA. More interestingly, we find the possible change at the first follow-up after the surgery of breast cancer. This change closely related to breast cancer surgery which is one of the most common causes of upper extremity lymphedema. We are recognized that the possible change may impact the development of lymphedema.

In the end of the manuscript, we revised this paragraph and we explain the reason in the Materials and methods part. **(Page 2, Paragraph 2)**

**9.-Table 1 clearly describes the sign differences between groups**

**10.-Tables 2-5 cannot be interpret based upon the table alone, additional information should be provided in a legend.**

**Response:** Thank you for your suggestion. we had provided additional information in the article and in a legend. Thank you. I learn a great deal from your propose. I have revised the content of this part.**(Table1-5)**

Special thanks to you for your good comments.

Response to Reviewer2: Thank you for your review of our paper. We have answered each of your points below.

1. **Although this is a very important topic, you seem to claim that this technique is novel, whereas there has been an extensive literature on the use of bioimpedance spectroscopy in the early detection of subclinical lymphoedema after breast cancer treatment (See examples of existing literature below). This has already addressed a number of issues which you raise as needing further exploration eg the effect of the dominant arm on bioimpedance values; the value of preoperative assessments followed by post operative measurements in demonstrating those who experience a change which then leads on to clinical lymphoedema; normative values and the use of a 2SD or 3SD cut offs (Fu 2013).**

**Response:** Thank you for your question. We read the literature you recommend carefully. We believe your advice will improve the paper significantly. I have reviewed the existing literature and then revised the contents of this article. In fact, this technique is not novel. Yet, the correlation between invasive cancer diagnosis, dissection/disruption of axillary lymph nodes and secondary lymphedema are not yet understood and assessed. It is core to our study. Moreover, there are many types of bio-impedance devices that are routinely used in clinical practice around the world. The absolute values therefore may not be directly comparable. So it's an exploratory experiment to make an attempt on setting up a new way to make data from different study comparable. **(Page 2, Paragraph 2)**

2. **page 8 “Subjects”: second paragraph: Why did you exclude patients who had radiotherapy or chemotherapy? Radiotherapy and taxane chemotherapy are known to be risk factors for the development of breast cancer related lymphoedema.**

**Response:** Thank you for your question. Dissection/disruption of axillary lymph nodes, radiotherapy and chemotherapy are the risk factors for the development of breast cancer related lymphedema. In our study, we did not evaluate all the risk of breast cancer related lymphedema, but only dissection/disruption of axillary lymph nodes. Therefore, we exclude patients who had radiotherapy or chemotherapy. These treatment will cause major effects on results. **(Page 5, Paragraph 2)**

3. **Page 9: it is not clear here when the data were collected. You quote “about” the time of surgery but this seems to be post operatively and in the window before chemotherapy or radiotherapy treatment is given?**

**Response:** Thank you for your question. I am very sorry that this part was not clear in the original manuscripts, I should have explain that surgical times were recorded as one of surgical data. And we chose the day before the data of first chemotherapy treatment to overcome the influence of chemotherapy. I have revised the content of this part.**(Page 5, Paragraph 2)**

4. **Page 9 evaluation criteria: In existing studies of the early detection of breast cancer related lymphoedema by limb volume measurements, a relative arm volume increase (compared with the contralateral arm) is commonly used. The two centimetres circumferential difference is on outmoded definition.**

**Response:** Thank you for your suggestion. In the article, our essay mentions the standard of 2cm- difference based on circumference. This standard is outdated. Your suggestion pointed out exactly where we had gone wrong. I have revised the contents of this part**(Page 7, Paragraph 1)**. Thank you very much.

5. **Page 9: the use of the term “screened patients” is not clear. Does it mean patients who fulfil the criteria of subclinical lymphoedema defined in your study?**

**Response:** Thank you for your question. I am very sorry that this part was not clear in the original manuscripts. The term “screened patients” is defined as patients who fulfil the criteria of subclinical lymphedema in our study. I will give a clear definition of this word in our article.**(Page 7, Paragraph 1)**

6. **Page 11 and 12: most of the existing literature uses the measurement of extracellular fluid (ECF) in the affected limb compared with the control arm, most commonly expressed as a ratio (L-Dex, in one manufacturer's device). This seems to that be validated by your study but you have also found some other measures which may be helpful. It will be important to compare these results with the existing literature and demonstrate if your other measures add anything to the existing knowledge.**

**Response:** Thank you for your suggestion. I believe that your suggestion is important and I'll follow any advice you give me. We added the part that compare our results with the existing literature.**(Page 15, Paragraph 2)** Thank you very much.

7. **page 14: I agree that a prospective multi centre study with a larger cohort would be helpful but you make no reference to the fact that these have been carried out using other bioimpedance devices for breast cancer related lymphedema.**

**Response:** Thank you for your question. The main risk factors for breast cancer-associated lymphedema (BCAL) include invasive cancer diagnosis, dissection/disruption of axillary lymph nodes, radiation therapy, local infection, and obesity, but other factors may also contribute. A prospective multi-centre study with a larger cohort would be helpful to assess the impact of different treatment related factors. We added the part that compare our results with the existing literature to explain the difference. **(Page 12-17)**

8. **In your references, reference 13 duplicates reference 10; reference 22 duplicates reference 16; reference 21 duplicates reference 20 and reference 23 duplicates reference 15.**

**Response:** Thank you for your question. We are very sorry for our negligence on references. I have revised the contents of this part. **(Page 20-23)**

9. **In the tables it would be important to define what is meant by “screened patients” as raised above under comments on page 9.**

**Response:** Thank you for your question. The term “screened patients” is defined as patients who fulfil the criteria of subclinical lymphedema in our study. I will give a clear definition of this word in the table.**(Page 7, Paragraph 1)**

We tried our best to improve the manuscript and made some changes in the manuscript. These changes will not influence the content and framework of the paper. And here we did not list the changes but marked in red in revised paper.

We appreciate for Editors/Reviewers’ warm work earnestly, and hope that the correction will meet with approval.

Once again, thank you very much for your comments and suggestions.
