# Supplementary material for: Bioelectrical impedance analysis for early screening of upper limb subclinical lymphedema: A case–control study
Source: PLoS One. 2022 Sep 19;17(9):e0274570. doi: 10.1371/journal.pone.0274570 (PMC9484636; doi:10.1371/journal.pone.0274570)
Supplement: Attachment — Submitted filename: Response to Reviewers.docx [file pone.0274570.s002.docx]

Dear Dr. Lafrenie

Subject: Submission of revised paper PONE-D-22-02659R1

Thank you for your email dated 27 July 2022 enclosing the reviewers’ comments. We have carefully reviewed the comments and have revised the manuscript accordingly. Our responses are given in a point-by-point manner below. Changes to the manuscript are shown in a separate file labeled “Revised manuscript with Track Changes”.

We hope the revised version is now suitable for publication and look forward to hearing from you in due course.

Sincerely,

Shaoyong Wu

International lymphedema therapist

West China Hospital

**Response to Reviewer 1: Thank you for your review of our paper. We have answered each of your points below.**

1. **Line 135-136 The authors now state that the 200ml or 10% vol diff are today's standard definition for BCRL. These definitions are also outdated. Today we use, 3% vol change corrected for body weight as a definition for early onset lymphedema. In patients suffering from edema we use <5% between 5-10% and >10% vol diff assessed either by water discplacment of volume calculation based upon circumference measuerements. In case of circumference measurements, measeurments are taken every 4 cm (4CM increments). The refs 30-33 that are used to motivate the 10% or 200ml are not approprioate as they are refs concerning BIA. Add the original research (and as mentioned in previous revies, ARMER et al have investigated these definitions). Additional refs that are lacking are: PMID: 33646139 and PMID: 33245458**

**Response:** Thank you for your suggestion. In the article, our essay mentions the standard of 200ml or 10% volume difference. This standard is outdated. Your suggestion pointed out exactly where we had gone wrong. I have revised the contents of this part.**(Line 135-137, 318-327)** Thank you very much.

I've read all the literature recommended by reviewer and then revised the contents of this article. **(Line 476-482)** We are very sorry for our negligence on references. Thank you very much.

2. **Line 96-97 is repetition of the eligibility criteria lines 102-105**

**Response:** Thanks very much for the suggestions from reviewer. I have revised the contents of this part.**(Line 96-97, 104)** Thank you very much.

3. **I still lack a piture of the BIA setup (preferably demonstrated on a patient)**

**Response:** Thank you for your question. As shown in the picture 1, we use InBody 770 multifrequency BI device (InBody 770, Cerritos, CA, USA) to measure and assess BIA of breast cancer survivors. Picture 2 present this BIA setup demonstrated on a patient.

**Picture 1 InBody 770, Cerritos, CA, USA**

**Picture 2	BIA setup (demonstrated on a patient)**

We tried our best to improve the manuscript and made some changes in the manuscript. These changes will not influence the content and framework of the paper. And here we did not list the changes but marked in red in revised paper.

We appreciate for Editors/Reviewers’ warm work earnestly, and hope that the correction will meet with approval.

Once again, thank you very much for your comments and suggestions.
